# The Benefits of Nanosized Magnesium Oxide in Fish *Megalobrama amblycephala*: Evidence in Growth Performance, Redox Defense, Glucose Metabolism, and Magnesium Homeostasis

**DOI:** 10.3390/antiox12071350

**Published:** 2023-06-27

**Authors:** Ling Zhang, Zishang Liu, Ying Deng, Chaofan He, Wenbin Liu, Xiangfei Li

**Affiliations:** Key Laboratory of Aquatic Nutrition and Feed Science of Jiangsu Province, College of Animal Science and Technology, Nanjing Agricultural University, No. 1 Weigang Road, Nanjing 210095, China

**Keywords:** magnesium oxide nanoparticles, redox defense, glucose metabolism, magnesium homeostasis, *Megalobrama amblycephala*

## Abstract

This study evaluated the effects of dietary magnesium oxide nanoparticles (MgO NPs) on the growth, redox defense, glucose metabolism, and magnesium homeostasis in blunt snout bream. Fish (12.42 ± 0.33 g) were fed seven diets containing graded levels of MgO NPs (0, 60, 120, 240, 480, 960, and 1920 mg/kg) for 12 weeks. Whole-body Mg retention decreased significantly as the dietary Mg increased. As dietary MgO NPs levels reached 120 mg/kg, the growth performance and feed utilization remarkably improved. When added at 240 mg/kg, oxidative stress was significantly reduced evidenced by the increased *Mn-sod* transcription and the decreased CAT and GSH-Px activities and the MDA content. Meanwhile, it enhanced glucose transport, glycolysis, and glycogen synthesis, while inhibiting gluconeogenesis, as was characterized by the increased transcriptions of *glut2*, *gk*, and *pk*, and the decreased transcriptions of *fbpase* and *g6pase*. In addition, the supplementation of 120 mg/kg MgO NPs promoted Mg transport marked by a significant increase in the protein expressions of TRMP7, S41A3, and CNNM1. In conclusion, the moderate supplementation of MgO NPs improved the growth performance, reduced hepatic oxidative stress, and promoted glucose transport, glycolysis, glycogen synthesis, and magnesium homeostasis in fish while inhibiting glu.

## 1. Introduction

Magnesium (Mg) is an essential element for fish. Generally, fish can obtain Mg from both water and feed with each species having a different dietary requirement [1]. Meanwhile, the Mg content of freshwater and seawater are significantly different, which is averaged at 5.6 and 1350 mg/L, respectively. Thus, unlike saltwater fish, freshwater fish cannot intake sufficient Mg to meet their needs in a freshwater environment [2]. For this reason, proactive Mg supplementation for them is extremely important, as is evidenced by the fact that diet provides 70–90% of the total Mg for freshwater fish [3]. However, dietary supplementation of Mg must be scientifically evaluated since inappropriate dietary Mg levels could cause several deleterious effects in fish, such as the loss of appetite, slow growth, sluggish activity, convulsions, vertebral curvature, and increased mortality [4,5,6].

Magnesium ion (Mg^2+^), the most abundant divalent cation in the cell, is closely involved in the intermediary metabolism by catalyzing or activating more than 300 enzymes [7,8]. Through this, Mg is involved in magnesium homeostasis, bone development, nucleic acid metabolism, antioxidant defense, and immunological reaction [9,10]. More importantly, imbalanced magnesium homeostasis has been demonstrated to be an important trigger for impaired glucose metabolism, since Mg acts as a cofactor for several glycolytic enzymes, such as hexokinase, phosphofructokinase, and pyruvate kinase, and also for several subunits of the electron transport chain, which is involved in the regulation of energy metabolism, carbohydrate oxidation, and glucose transport [11,12]. To date, several studies have revealed that insufficient Mg intake and low serum Mg concentration may contribute to insulin resistance and type 2 diabetes, as well as the associated metabolic syndromes [13,14,15]. For example, type 2 diabetes mellitus is often associated with hypomagnesemia and cellular Mg^2+^ loss [15]. In addition, an imbalanced intracellular Mg^2+^ transport was observed in the cardiomyocytes of diabetic rats, thereby leading to an imbalance in cellular Mg^2+^ homeostasis [16]. However, the previous research mainly focuses on mammals with relevant information being scarce, in aquatic species. In fish, Wei et al. have found that Mg inhibits lipogenesis, and increases lipolysis, which in turn ultimately reduces lipid deposits in the liver of yellow catfish (*Pelteobagrus fulvidraco*) [17]. In addition, Bao et al. have demonstrated that the growth performance and muscle development of blunt snout bream (*Megalobrama amblycephala*) were both enhanced by Mg supplementation when offered a high carbohydrate diet [18]. However, the molecular events underlying these effects are still poorly elucidated. Whether they are fulfilled through the promoted redox defense and magnesium homeostasis is still unknown.

In the wake of the rapid development of nanotechnology, magnesium oxide nanoparticles (MgO NPs) have been used in animal nutrition with novel characteristics, such as notably excellent surface activity, considerable catalytic coefficient, high adsorption capacity as well as minimal toxicity [19,20]. Additionally, MgO NPs are also of great interest to researchers due to their excellent ability to enhance the redox defense, and to treat diabetes [21]. For example, MgO NPs can decrease the serum glucose in a diabetic rat [22], and reverse the insulin resistance in diabetic c3 T3-L1 adipocytes [23]. In addition, enhanced catalase and superoxide dismutase activities were also observed in a legume plant (horse gram, *Macrotyloma uniflorum*) [24] as well as in mice infected with sporulated oocysts after the treatment of MgO NPs [25]. In fish, a previous study has reported that dietary magnesium oxide nanoparticles and selenium oxide nanoparticles have synergistic effects in Asian black bass (*Lates calcarifer*), as was revealed by the improved growth, increased digestive enzyme activity, and enhanced immunity [19]. However, the potential of MgO NPs for bio-applications has not yet been completely explored within the realm of nanoparticles, especially in the aquaculture specialty.

Blunt snout bream is one of the most extensively farmed freshwater fish in the Chinese intensive aquaculture system with an annual production of 781,737 tons in 2020 [26]. This species has a flat, high, and rhombus-shaped body with a greenish-grey color. Due to its herbivorous character, successful artificial breeding, high disease resistance, fast growth rate, and relatively low cost, this species has a high market prospect in China [27]. Regarded as a great delicacy, the demand for its product is steadily increasing. This study evaluated the optimum Mg requirement of this species using MgO NPs as a new Mg source. In addition, the effects on antioxidant capacity, glucose metabolism, and magnesium homeostasis were also investigated to further evaluate the effectiveness of MgO NPs as a new feed additive in aquaculture.

## 2. Materials and Methods

### 2.1. Fish, Diet, and the Experimental Procedure

The research was implemented in an indoor recirculating water system at the aquaculture base of Nanjing Agricultural University. Juvenile *M. amblycephala* (12.42 ± 0.33 g) were assigned randomly to 28 tanks with 14 fish per tank. Then, a total of seven diets were prepared with 0, 60, 120, 240, 480, 960, and 1920 mg/kg dietary MgO NPs (item no. 1309-48-4, Aladdin, Shanghai, China) added in the basal diet, respectively (Table 1). Among them, fish meal, casein, and gelatin are the protein sources, corn starch is the carbohydrate source, and fish oil and soybean oil in equal amounts are the fat source. The feed ingredients were weighed and mixed proportionally. Trace ingredients such as MgO NPs and premix were mixed using the stepwise expansion method. Then, fish oil, soybean oil, and water were added in the corresponding ratios. After mixing all the ingredients, the feed was made into long strips with a diameter of 2.50 mm by a twin-screw extruder. The experimental feed was later dried, crushed, and stored at −20 °C. As determined by the inductively coupled plasma-emission spectroscopy, the content of Mg in the experimental feed was 26.03, 77.11, 149.02, 229.00, 501.13, 1090.32, and 2083.05 mg/kg, respectively. Fish were fed to visual satiety three times daily for 12 weeks, and four replicates were set for each diet. Dead fish were removed daily, and the ration size was adjusted taking into consideration the influence of mortality. The initial whole-body Mg content of *M. amblycephala* was 5.72–6.46 mg/kg. Water quality was monitored as follows: water temperature 27.5–29.6 °C, dissolved oxygen > 6.0 mg/L, and pH 7.4–7.7. Regular monitoring of the Mg concentration in the incubation water was also conducted during the trial, with a concentration of 8.1 mg/L Mg.

### 2.2. Sample Collection

Following the feeding trial, a 24-h fast was employed to empty the intestinal contents of the fish. Then, the number and weight of fish in each tank were recorded separately. Four fish were collected randomly in each tank and anesthetized with 100 mg/L MS-222 (Solarbio, Beijing, China). Blood was collected from the tail vein of the fish with a 2 mL sterile heparinized syringe and centrifuged at 3 × 10^3^ r/min for 8 min at 4 °C to obtain the plasma. Following this, livers were collected and weighed. All samples are frozen in liquid nitrogen and refrigerated at −80 °C.

### 2.3. Analysis of Diets and Body Composition

A flame atomic absorption spectrophotometric method was used to measure the Mg content in diet and fish. Additionally, the nutrient content of the diet was assayed according to the standard method detailed by AOAC [28]. Specifically: (1) the diets and fish were weighed, then baked at 105 °C to a constant weight with the moisture content calculated; (2) the total nitrogen content was determined using an automatic Kjeldahl nitrogen tester (FOSS KT260, Switzerland), and then multiplied by 6.25 to calculate the crude protein content; (3) after the addition of ether to the Soxhlet extractor, the pre-weighed fat pack containing the samples was refluxed at 65–70 °C for 12 h. Then, the samples were dried to a constant weight and were weighed with the fat content calculated; (4) the samples were subjected to acid and alkaline treatment, and the crude fiber content of the feed was determined using a fully automated fiber analyzer (ANKOM A2000 i, New York, NY, USA); (5) the samples were slightly burned in an electric furnace, then burnt in a muffle furnace at 550 ± 20 °C for 4–6 h, and then cooled overnight. The crude ash content was calculated by weighing; (6) the total energy of the feeds was determined using an oxygen bomb calorimeter (PARR 1281, Moline, IL, USA).

### 2.4. Analysis of Plasma Metabolites

Plasma glucose (GLU), glycated serum proteins (GSP), total cholesterol (TCHO), triacylglycerol (TRIG), and Mg levels were all measured by a Hitachi P-module automated chemical analyzer (Roche Diagnostics, Indianapolis, IN, USA) in random order.

### 2.5. Analysis of the Oxidative Stress-Related Parameters

According to the method of Aebi [29], the activity of catalase (CAT) was estimated by observing the rate of decomposition of H_2_O_2_ by measuring the decrease in absorbance at 240 nm, and 1 μmol H_2_O_2_ consumed per mg of protein per minute at 25 °C is regarded as a CAT activity unit. Total superoxide dismutase (SOD) activity was measured according to the method of Marklund and Marklund [30], and the activity is given in SOD units (1 SOD unit = 50% inhibition of the xanthine oxidase reaction) per mg of protein at 25 °C. Likewise, the activity of glutathione peroxidase (GSH-Px) was determined using the Paglia and Valentine method [31]. The enzyme activity was calculated using an extinction coefficient (6.22 × 10^3^ M^−1^ cm^−1^) of nicotinamide adenine dinucleotide phosphate (NADPH), and 1 unit enzyme activity was defined as 1 nmol of NADPH oxidized per minute/mg of protein at 25 °C. In addition, the content of malondialdehyde (MDA) was assayed by the thiobarbituric acid (TBA) method [32]. Briefly, the hepatic homogenate was incubated with 100% trichloroacetic acid, and the supernatant was measured spectrophotometrically at 532 nm after centrifugation and was incubated at 100 °C for 30 min with 1% TBA, 0.05 mol/L NaOH, and 0.025 mM butylhydroxytoluene. The soluble protein concentration of liver homogenates was measured following the method of Bradford [33].

### 2.6. RNA Isolation and Quantitative Real-Time-PCR

Following the instructions of the total RNA extraction (DNase I) kit (AG12001, Accurate biology, Changsha, China), total RNA was extracted from the livers of four fish within each group. Reverse transcription was performed using the mRNA cDNA synthesis kit (AG11705, Accurate Biology, Changsha, China). The premix with a volume of 20 μL (containing 1 μg of RNA) was prepared. The mixture was incubated at 42 °C for 2 min and 37 °C for 15 min, and then at 85 °C for 5 s. Upon completion of the reaction, the mixture was centrifuged with the solution collected. The cDNA synthesized by the reverse transcription was stored at −80 °C. Amplification was carried out using the mRNA qPCR kit in accordance with the manufacturer’s instructions. The amplification process was as follows: 95 °C for 30 s, then heated to 95 °C for 5 s, cooled to 60 °C for 30 s, and cycled 40 times. The melting curve was analyzed at 60 °C. The cDNA in each sample was diluted 10-fold to 2 μL as a template with the target gene primers and internal reference gene primers added separately for amplification. Melting point curves were analyzed at temperatures between 60 and 95 °C. The mRNA expression of genes was calculated using the 2^−ΔΔCT^ method with *ef1*-*α* as the internal reference. Primer designs for the genes are shown in Table 2.

### 2.7. The Western Blotting Assay

Cell lysates were prepared by incubating with a radio immunoprecipitation assay lysis buffer containing protease inhibitors. The proteins in the lysate were separated by the 4–20% precast protein plus gel (Cat#36250 ES10, Yeasen Biotechnology Co., Ltd., Shanghai, China) electrophoresis, and were transferred onto polyvinylidene fluoride membranes. Next, the membranes were blocked for 15 min in a fast blocking solution (36122 ES60, Yeasen Biotechnology, Shanghai, China), and were incubated with primary antibodies overnight at 4 °C. Anti-β-ACTIN (42 KDa, 1:1000, 66009-1-Ig, Proteintech, Wuhan, China), anti-TRMP7 (217 KDa, 1:1000, AF301587, AiFang Biological, Changsha, China), anti-S41A3 (56 KDa, 1:1000, AF14148, AiFang Biological, Changsha, China), and anti-CNNM1 (104 KDa, 1:1000, AF09406, AiFang Biological, Changsha, China) were all used. After washing three times with TBST, the membranes were incubated with secondary antibodies (1:5000, BA1054, Boster, Wuhan, China) for 2 h. The immunoreactive bands were detected using a high-sensitivity chemiluminescence kit (E412-01/02, Vazyme, Nanjing, China). Then β-ACTIN was used to normalize the protein expressions, and the bands were visualized and quantified using the Image J V1.8.0.112 software.

### 2.8. Statistical Analysis

Data were analyzed using the SPSS 25.0 software, and one-way ANOVA was conducted after testing the homogeneity of variance using Levene’s test [35]. If there were differences in the statistics, Turkey’s multiple range test was then performed for multiple comparisons. In addition, all data were compared with orthogonal polynomial contrasts to determine whether the effect was linear, quadratic, or cubic. All results were expressed as mean ± standard error, and the significance level was set at *p* < 0.05. In addition, broken-line regression and power function analysis were used to determine the optimum Mg requirement [36].

## 3. Results

### 3.1. Growth Performance

**Figure 1:**
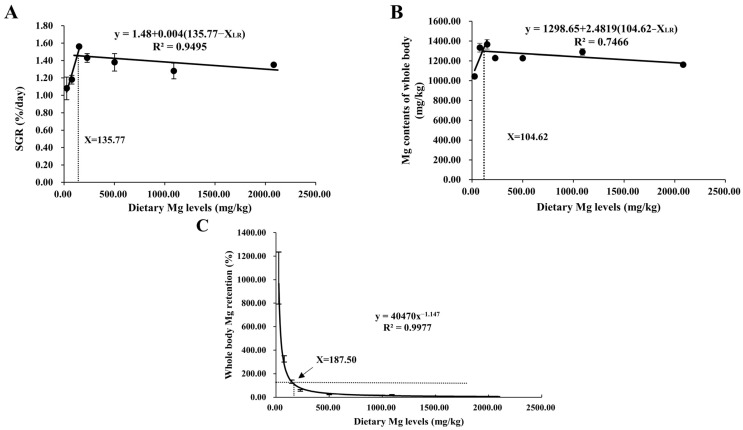
Relationship between dietary Mg levels (mg/kg) and SGR (%/day) (**A**), and whole-body Mg content (mg/kg) (**B**), and Mg retention (%) (**C**) of *M. amblycephala*, respectively.

As shown in Table 3, final weight (FW), specific growth rate (SGR), survival rate (SR), and protein efficiency ratio (PER) all increased quadratically (*p* < 0.05) with increasing dietary MgO NPs contents from 0 to 120 mg/kg. Then SR and PER both decreased significantly with further increases in MgO NPs levels, while FW and SGR both plateaued (*p* > 0.05). Unlikely, the opposite result was observed in FCR. However, feed intake (FI) increased linearly (*p* < 0.001) as dietary MgO NPs levels increased from 0 to 1920 mg/kg. As shown in Figure 1, the relationship between SGR and whole-body Mg content against dietary Mg levels was represented by a broken line regression model, indicating that the optimum dietary Mg requirement was 135.77 and 104.62 mg/kg diet, respectively. In addition, Mg requirements were estimated for *M. amblycephala*. Dietary Mg of 187.50 mg/kg was a concentration between the whole-body Mg retention and the dietary Mg levels when the whole-body Mg retention was 100%.

### 3.2. Body Composition Analysis

As we can see from Table 4, little difference (*p* > 0.05) was found in the content of moisture, crude lipid, and ash among all the treatments. The content of crude protein increased quadratically (*p* < 0.05) as dietary MgO NPs levels increased from 0 to 120 mg/kg, and then plateaued (*p* > 0.05) with further increasing MgO NPs levels. Whole body Mg content increased both quadratically and cubically (*p* < 0.001) as dietary MgO NPs levels increased from 0 to 120 mg/kg, then decreased sharply (*p* < 0.05) with further increasing MgO NPs levels. In addition, whole-body Mg retention decreased linearly, quadratically, and Cubically (*p* < 0.05) as the dietary Mg levels increased.

### 3.3. Plasma Biochemical Parameters

As we can see from Table 5, little difference (*p* > 0.05) was found in the concentration of TCHO among all the treatments. The concentration of GLU, GSP, and TRIG all decreased remarkably (*p* < 0.05) as dietary MgO NPs levels increased from 0 to 240 mg/kg. Then they all increased when dietary MgO NPs levels reached 960 mg/kg, but decreased with further increasing MgO NPs levels. However, plasma Mg concentration increased linearly, quadratically, and cubically (*p* < 0.05) with the increase in dietary MgO NPs levels, and maximized at the 960 mg/kg group, then decreased sharply (*p* < 0.05) with further increasing MgO NPs levels.

### 3.4. Antioxidant Enzyme Activities and the MDA Content

**Figure 2:**
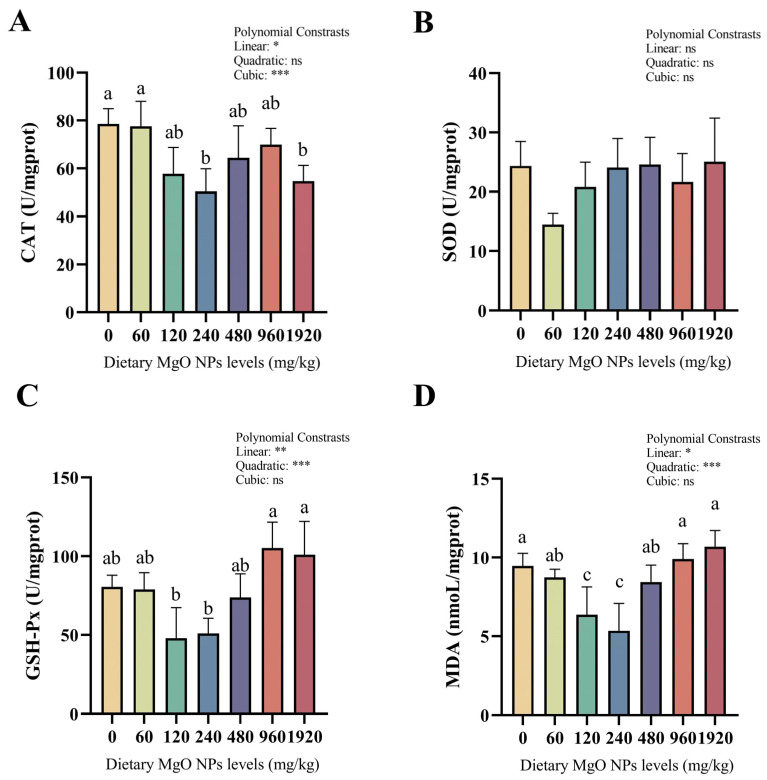
Hepatic CAT (**A**), SOD (**B**), and GSH-Px (**C**) activities as well as MDA (**D**) contents. CAT, catalase; SOD, superoxide dismutase; GSH-Px, glutathione peroxidase; MDA, malondialdehyde. Each data point represents the mean of four replicates. Bars assigned with different superscripts are significantly different (*p* < 0.05). * *p* < 0.05, ** *p* < 0.01, *** *p* < 0.001, ns: no significance.

As we can see from Figure 2, the activity of SOD showed no significant difference among the different treatments. However, significant (*p* < 0.05) linear and quadratic effects were both observed in GSH-Px activities and MDA contents. CAT activities and MDA contents both decreased remarkably (*p* < 0.05) as dietary MgO NPs levels reached 240 mg/kg. MDA contents increased sharply (*p* < 0.05) with further increasing MgO NPs levels, while CAT activities plateaued (*p* > 0.05). In addition, GSH-Px activities decreased slightly (*p* > 0.05) as dietary MgO NPs levels reached 240 mg/kg, but increased sharply (*p* < 0.01) with further increasing MgO NPs levels.

### 3.5. Redox Defense and Glucose Metabolism-Related Gene Expression

**Figure 3:**
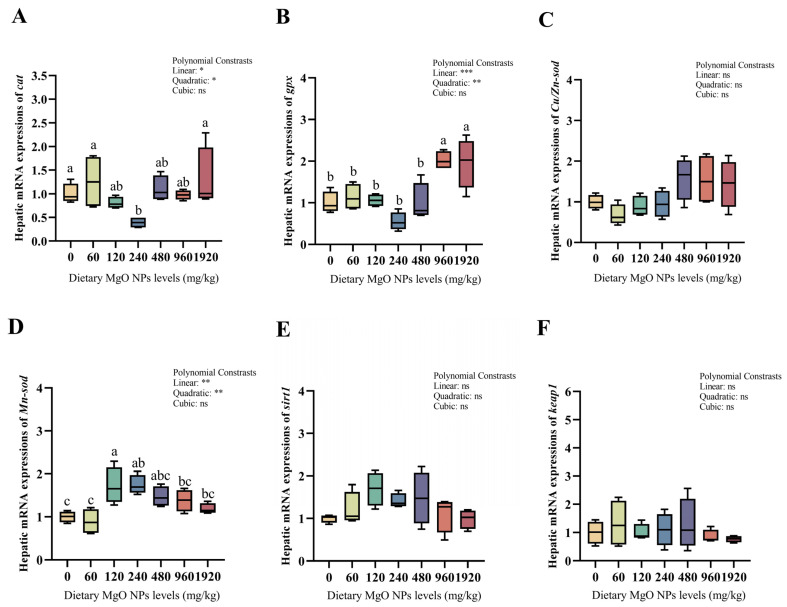
Hepatic transcriptions of the genes involved in the antioxidant defense. The mRNA levels of catalase (*cat*) (**A**), glutathione peroxidase (*gpx*) (**B**), copper/zinc superoxide dismutase (*Cu/Zn-sod*) (**C**), manganese superoxide dismutase (*Mn-sod*) (**D**), sirtuin 1 (*sirt1*) (**E**), and kelch-like ECH associating protein 1 (*keap1*) (**F**) were all evaluated using real-time RT-PCR. Data were normalized by *ef1*-*α*, and were referred to the values (relative units) found in fish fed 0 mg/kg MgO NPs. Each data point represents the mean of four replicates. Bars assigned with different superscripts are significantly different (*p* < 0.05). * *p* < 0.05, ** *p* < 0.01, *** *p* < 0.001, ns: no significance.

**Figure 4:**
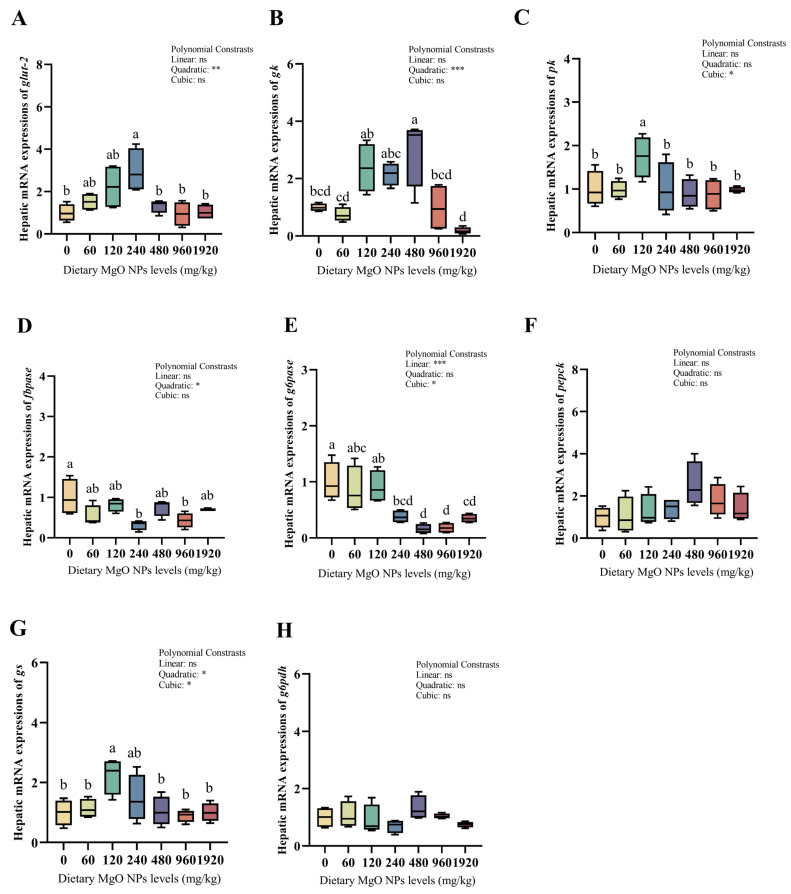
Relative transcriptions of the genes involved in glucose metabolism in the liver. The mRNA levels of glucose transporter 2 (*glut-2*) (**A**), glucokinase (*gk*) (**B**), pyruvate kinase (*pk*) (**C**), fructose-1,6-biphosphatase (*fbpase*) (**D**), glucose-6-phosphatase (*g6pase*) (**E**), phosphoenolpyruvate carboxykinase (*pepck*) (**F**), glycogen synthase (*gs*) (**G**), and glucose-6-phosphate dehydrogenase (*g6pdh*) (**H**) were all evaluated using RT-PCR. Data were normalized by *ef1*-*α*, and were referred to the values (relative units) found in fish fed 0 mg/kg MgO NPs. Each data point represents the mean of four replicates. Bars assigned with different superscripts are significantly different (*p* < 0.05). * *p* < 0.05, ** *p* < 0.01, *** *p* < 0.001, ns: no significance.

As we can see from Figure 3 and Figure 4, dietary MgO NPs levels exerted little effect on the transcriptions of *Cu/Zn-sod*, *sirt1*, *keap1*, *pepck,* and *g6pdh* (*p* > 0.05). The transcriptions of *Mn-sod*, *pk*, and *gs* all increased significantly (*p* < 0.01) with increasing dietary MgO NPs levels up to 120 mg/kg, and then decreased significantly (*p* < 0.05) with further increasing MgO NPs levels. The transcriptions of *glut-2* and *gk* both showed a similar trend and maximized at 240 and 480 mg/kg, respectively. However, the transcriptions of *cat*, *gpx*, *fbpase*, and *g6pase* all decreased significantly (*p* < 0.05) as dietary MgO NPs levels increased from 0 to 240 mg/kg. Then, the transcriptions of *cat* and *gpx* both increased significantly (*p* < 0.05) with further increasing MgO NPs levels, while those of *fbpase* and *g6pase* plateaued (*p* > 0.05).

### 3.6. Magnesium Homeostasis-Related Protein and Gene Expression

**Figure 5:**
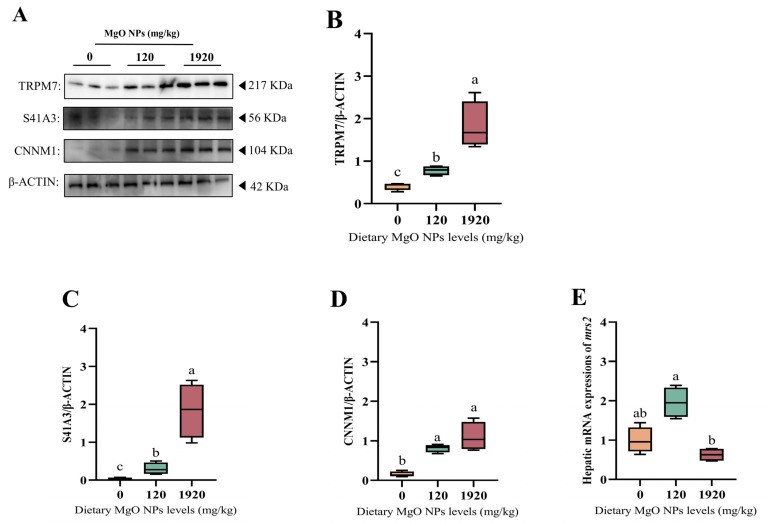
Hepatic expressions of the proteins and genes involved in magnesium homeostasis. (**A**–**D**) Western blot was performed to analyze the protein expression levels of transient receptor potential cation channel member 7 (TRMP7), solute carrier family 41 member 3 (S41A3), and ancient conserved domain-containing protein 1 (CNNM1), while (**E**) RT-PCR was performed to evaluate the transcription of mitochondrial RNA splicing 2 (*mrs2*). For protein expression, β-ACTIN was used to normalize the data. For gene expression, data were normalized by *ef1*-*α*, and were referred to the values (relative units) found in fish fed 0 mg/kg MgO NPs. Each data point represents the mean of four replicates. Bars assigned with different superscripts are significantly different (*p* < 0.05).

As exhibited in Figure 5, the protein expressions of TRPM7, S41A3, and CNNM1 were all up-regulated significantly (*p* < 0.05) with increasing dietary MgO NPs levels. The *mrs2* transcription of the 120 mg/kg group was significantly (*p* < 0.05) higher than that of the 1920 mg/kg group, but exerted little difference (*p* > 0.05) with that of the control group.

## 4. Discussion

In this study, Mg deficiency (0–60 mg/kg MgO NPs) impaired the growth performance of blunt snout bream as was evidenced by the decreased FW, SGR, and PER. Similarly, poor growth performance and feed efficiency were also observed in other aquatic animals offered Mg-deficient diets [1,37,38,39]. However, the growth performance of fish fed 120–140 mg/kg MgO NPs was significantly higher than those of the other groups. This may be attributed to the fact that appropriate Mg supplementation could enhance the activities of intestinal digestive and brush border enzymes in fish, thus boosting nutrient utilization and feed efficiency [40]. Similarly, the growth rate of yellow catfish and common carp (*Cyprinus carpio*, L.) both increased when offered appropriate Mg levels [17,41]. The optimal Mg requirement of *M. amblycephala* was estimated to be 104.62–187.50 mg/kg by analysis of SGR, whole-body Mg content, and Mg retention against different dietary Mg levels. In comparison, it is lower than those of other fish, such as Atlantic salmon (*Salmo salar*), grass carp (*Ctenopharyngodon idella*), and common carp (*Cyprinus carpio* var.), whose dietary magnesium requirement is 330, 690, and 800 mg/kg, respectively [42]. This discrepancy may be attributed to the fact that (1) the Mg requirement of fish is closely dependent on the differences in species, feeding habits, and feed types [43]; and (2) compared with other Mg sources, MgO NPs have a high bioavailability due to its smaller size and easier absorption into the intestinal wall [19,20]. Furthermore, it was also found in this study that excessive Mg supplementation significantly inhibited the growth performance of fish. The speculation is that high Mg intakes might impair gastrointestinal function [44]. In addition, whole-body crude protein content was significantly elevated when dietary MgO NPs levels increased from 0 to 120 mg/kg. Actually, Mg is involved in protein synthesis through the engagement in amino acid activation and the attachment of mRNA to ribosomes [45]. This might be responsible for the relatively low body protein deposition in fish fed the Mg-deficient diets in this study. In line with our study, Wang et al. have also revealed that the supplementation of moderate amounts of Mg sulfate in feeds greatly elevated the whole-body crude protein content of grass carp [1].

Mg has been shown to be a key factor in blood glucose control. The results presented in this experiment showed that dietary MgO NPs supplementation had a positive effect on reducing plasma glucose concentrations. This effect may be fulfilled via the regulation of the oxidative phosphorylation and glycolysis pathways [46]. In addition, the activation of insulin receptor tyrosine kinase requires binding to Mg^2+^, which in turn enables glucose and insulin metabolism [47]. In addition, Mg modulates the translocation of glucose into the cell, thus governing the extracellular glucose levels [48]. Plasma GSP levels in this study further supported this result, as it is regarded as an accurate indicator of hyperglycemic status [49]. In the present study, MgO NPs supplementation lowered plasma TRIG concentration but did not significantly alter the concentration of TCHO. Similar to our results, Corica et al. have also demonstrated that oral Mg supplementation reduced lipid concentrations in diabetic patients [50]. In addition, Gueux et al. have found that Mg deficiency increased lipid levels in rats by decreasing the clearance of circulating lipids [51]. Thus, the decrease in plasma TG concentration in the present study may be a result of an increased clearance of circulating lipids due to Mg supplementation, which deserves further studies.

In the biological organism, various antioxidant defense systems have evolved. GSH-Px is a widely available peroxidolytic enzyme, whereas SOD and CAT are scavengers of reactive oxygen radicals. In addition, MDA is the product of lipid peroxidation that serves as an important indicator of free radical-induced membrane damage [52]. In the current study, hepatic CAT activities and MDA contents were significantly lower in the 120 and 240 mg/kg MgO NPs groups compared with other treatments, indicating that appropriate Mg levels reduced oxidative stress. This was further supported by the up-regulated transcriptions of both *cat* and *gpx*. This observation is confirmed by Wang et al., who have documented that grass carp was vulnerable to lipid peroxidation when fed Mg-unbalanced diets [1]. In addition, Mg can decrease the infarct size in patients with acute myocardial infarction by reducing the generation of free radicals [49], and also attenuating the hyperglycemia-induced damage by suppressing the formation of reactive oxygen species [53]. As such, a similar mechanism could be presented in blunt snout bream. More importantly, MnSOD, a manganese metalloenzyme, has been identified as a key regulator of oxidative stress [54], which inhibits the accumulation of oxidative stress by reducing superoxide. It has been confirmed that the enzymes activated by manganese can also be activated by other metals, particularly Mg [55,56]. Therefore, this might be advanced to interpret the increased transcription of *Mn-sod* in the 120 and 240 mg/kg MgO NPs groups in this study. Together, the results suggested that an appropriate Mg supplementation reduced the hepatic oxidative stress in fish.

As a cofactor of several enzymes implicated in glucose metabolism, Mg plays an imperative role in maintaining glucose homeostasis. In this study, dietary supplementation of MgO NPs up to 120–240 mg/kg increased the transcriptions of *glut2*, *gk,* and *pk*, but decreased those of *fbpase* and *g6pase*, suggesting that MgO NPs could improve hepatic glucose transport, glycolysis, and glycogen synthesis in fish while inhibiting gluconeogenesis. Generally, after passing into the pancreatic beta cells in the presence of GLUT-2, glucose is converted by GK to G6P, which is further processed to generate ATP [57]. Therefore, the GLUT-2 and GK tandem is generally referred to as a glucose sensor that regulates blood glucose levels. Supportively, altering the glucose-dependent activity of GK by mutations can dramatically affect the set point of systemic glucose homeostasis [58,59]. In addition, many glycolytic enzymes including GK have a requirement for Mg^2+^ and utilize adenine nucleotides (MgATP) as a cofactor [60,61]. This might be advanced to interpret the increased transcriptions of *glut2*, *gk*, and *pk*. In addition, hepatic isozymes of FBPase have been implicated as regulatory enzymes of gluconeogenesis [62]. Previously Mg has been demonstrated to improve the sensitivity of insulin, which destroys glucose generation by preventing the activities of gluconeogenic enzymes [63].

Considering the important effects of dietary Mg supplementation on Mg status, we further selected the Mg-deficient, moderate, and excess groups (namely the 0, 120, and 1920 mg/kg MgO NPs treatments) to explore the effects of MgO NPs on the magnesium homeostasis of fish. In this study, the protein expression of the Mg^2+^ influx flow gene TRMP7 (transient receptor potential melastatin-subfamily member 7) and the Mg^2+^ efflux-related gene S41A3 (solute carrier family 41 member 3) and CNNM1 (ancient conserved domain-containing protein 1) were significantly reduced with increasing doses of MgO NPs, while the transcription levels of the mitochondrial Mg^2+^ inward flow transporter *mrs2* (mitochondrial RNA splicing 2) showed an increasing trend followed by a significant reduction. This suggests that MgO NPs can enter the liver tissue of *M. amblycephala* via the Mg^2+^ transport or endocytosis pathway. Meanwhile, either excessively low or high MgO NPs can contribute to an imbalanced magnesium homeostasis. Supportively, magnesium homeostasis has been reported to be maintained by many hormones and specific transporters. Particularly, TRPM7 mediates the ubiquitous Mg uptake at the cellular/tissue level [64], while both S41A3 and CNNM1 are responsible for the Mg^2+^ efflux [65]. Additionally, magnesium homeostasis is maintained by mrs2, a significant mitochondrial mg^2+^ transporter [66].

## 5. Conclusions

In general, the moderate supplementation of MgO NPs improved the growth performance, reduced hepatic oxidative stress, and promoted glucose transport, glycolysis, glycogen synthesis, and magnesium transport in blunt snout bream while inhibiting gluconeogenesis. The broken-line regression analysis of SGR and whole-body Mg content revealed that the appropriate Mg requirement for this species was 104.62–187.50 mg/kg.

## Figures and Tables

**Table 1:** Formulation and proximate composition of the basal diet.

Formulation	Contents (%)	Proximate Analysis	Contents
Fish meal	3	Moisture (%)	9.54
Casein	30	Crude protein (%)	31.16
Gelatin	7.5	Ether extract (%)	6.86
Corn starch	38.3	Crude fiber (%)	14.76
Fish oil	3.1	Ash (%)	3.49
Soybean oil	3.1	Energy (MJ/kg)	20.73
Cellulose	10		
Premix without magnesium	1.2		
Calcium biphosphate	1.8		
Carboxymethyl cellulose	2		

Premix supplied the following minerals and/or vitamins (per kg): CuSO_4_·5 H_2_O, 2.0 g; FeSO_4_·7 H_2_O, 25 g; ZnSO_4_·7 H_2_O, 22 g; MnSO_4_·4 H_2_O, 7 g; Na_2_SeO_3_, 0.04 g; KI, 0.026 g; CoCl_2_·6 H_2_O, 0.1 g; Vitamin A, 900,000 IU; Vitamin D, 200,000 IU; Vitamin E, 4500 mg; Vitamin K_3_, 220 mg; Vitamin B_1_, 320 mg; Vitamin B_2_, 1090 mg; Vitamin B_5_, 2000 mg; Vitamin B_6_, 500 mg; Vitamin B_12_, 1.6 mg; Vitamin C, 5000 mg; Folic acid, 165 mg; Choline, 60,000 mg; Myoinositol, 15,000 mg; Niacin, 2800 mg.

**Table 2:** Primer sequences used to assay gene expressions by real-time PCR.

Target Genes	Forward (5′-3′)	Reverse (5′-3′)	Accession Numbers or Reference
*glut-2*	ACGCACCCGATGTGAAAGT	TTGGACAGCAGCATTGATT	KC513421.1
*gk*	AAAATGCTGCCCACTTAT	AATGCCCTTATCCAAATC	KJ141202.1
*pk*	GCCGAGAAAGTCTTCATCGCACAG	CGTCCAGAACCGCATTAGCCAC	[34]
*fbpase*	TACCCAGATGTCACAGAAT	CACTCATACAACAGCCTCA	KJ743995.1
*g* *6pase*	TGAGACCCGGTTTTATGGAG	CATGCAGACCACCAGCTCTA	[34]
*pepck*	TGGCCCGTGTGGAGAGTAAAA	ATGTGTTCTGCCAGCCAG	[34]
*gs*	CCTCCAGTAACAACTCACAACA	CAGATAGATTGGTGGTTACGC	[34]
*g* *6pdh*	AGGTAAAGGTGCTGAAGT	AAATGTAGCCTGAGTGGA	KJ743994.1
*cat*	CAGTGCTCCTGATACCCAGC	TTCTGACACAGACGCTCTCG	XM_048158628.1
*Cu/Zn-sod*	AGTTGCCATGTGCACTTTTCT	AGGTGCTAGTCGAGTGTTAGG	KF479046.1
*Mn-sod*	AGCTGCACCACAGCAAGCAC	TCCTCCACCATTCGGTGACA	KF195932.1
*gpx*	GAACGCCCACCCTCTGTTTG	CGATGTCATTCCGGTTCACG	KF378713.1
*sirt1*	TCGGTTCATTCAGCAGCACA	ATGATGATCTGCCACAGCGT	MT518159.1
*keap1*	AATATCCGCCGGCTGTGTAG	TGAGTCCGAGGTGTTTCGTG	XM_048200093.1
*ef1-α*	CTTCTCAGGCTGACTGTGC	CCGCTAGCATTACCCTCC	X77689.1

*glut-2*, glucose transporter 2; *gk*, glucokinase; *fbpase*, fructose-1,6-biphosphatase; *g6pase*, glucose-6-phosphatase; *pk*, pyruvate kinase; *pepck*, phosphoenolpyruvate carboxykinase; *gs*, glycogen synthase; *g6pdh*, glucose-6-phosphate dehydrogenase; *cat*, catalase; *Cu/Zn-sod*, copper/zinc superoxide dismutase; *Mn-sod*, manganese superoxide dismutase; *gpx*, glutathione peroxidase; *sirt1*, sirtuin 1; *keap1*, kelch-like ECH associating protein 1; *ef1-α*, elongation factor 1 α.

**Table 3:** Growth performance and feed utilization of *M. amblycephala* fed the experimental diets.

Dietary MgO NPs Levels (mg/kg)	IW (g)	SR (%)	FW (g)	SGR (%/d)	FI (g per Fish)	FCR	PER
0	12.14 ± 0.22	57.14 ± 4.12 ^b^	30.45 ± 3.67 ^c^	1.08 ± 0.13 ^b^	46.40 ± 8.29 ^c^	2.55 ± 0.04 ^b^	1.97 ± 0.13 ^ab^
60	12.24 ± 0.29	71.43 ± 8.25 ^ab^	32.92 ± 1.55 ^bc^	1.18 ± 0.05 ^b^	51.15 ± 2.41 ^c^	2.48 ± 0.06 ^b^	1.91 ± 0.01 ^ab^
120	12.57 ± 0.22	90.48 ± 2.38 ^a^	46.45 ± 2.01 ^a^	1.56 ± 0.02 ^a^	61.79 ± 2.84 ^bc^	1.83 ± 0.10 ^c^	2.24 ± 0.12 ^a^
240	12.48 ± 0.05	88.10 ± 2.38 ^a^	41.56 ± 2.82 ^ab^	1.43 ± 0.05 ^ab^	81.48 ± 3.42 ^ab^	2.83 ± 0.24 ^b^	1.51 ± 0.11 ^bc^
480	12.38 ± 0.27	83.33 ± 6.30 ^a^	39.77 ± 2.59 ^abc^	1.38 ± 0.10 ^ab^	68.61 ± 5.87 ^bc^	2.51 ± 0.06 ^b^	1.72 ± 0.04 ^b^
960	12.57 ± 0.08	59.52 ± 2.38 ^b^	36.97 ± 4.26 ^abc^	1.28 ± 0.09 ^ab^	67.95 ± 9.88 ^bc^	2.76 ± 0.14 ^b^	1.61 ± 0.14 ^bc^
1920	12.57 ± 0.14	57.14 ± 4.12 ^b^	38.95 ± 0.58 ^abc^	1.35 ± 0.01 ^ab^	97.70 ± 4.38 ^a^	3.70 ± 0.18 ^a^	1.20 ± 0.06 ^c^
Polynomial contrasts							
Linear	ns	ns	*	*	***	***	***
Quadratic	ns	***	**	**	ns	***	*
Cubic	ns	ns	ns	ns	ns	ns	ns

Survival rate (SR, %) = 100 × (final fish number/initial fish number). Specific growth rate (SGR, %/day) = (LnW_t_ − LnW_0_) × 100/T, where W_0_ and W_t_ are the initial and final body weights, and T is the culture period in days. Feed intake (FI, g per fish) = total feed intake (g)/total fish number. Feed conversion ratio (FCR) = feed consumption (g)/fish weight gain (g). Protein efficiency ratio (PER) = fish weight gain/total protein fed. Each data point represents the mean of four replicates. Means in the same line with different superscripts indicate a significant difference (*p* < 0.05). * *p* < 0.05, ** *p* < 0.01, *** *p* < 0.001, ns: no significance.

**Table 4:** Proximate composition and Mg contents of whole-body of *M. amblycephala* fed the experimental diets.

Dietary MgO NPs Levels (mg/kg)	Moisture (%)	Crude Protein (%)	Crude Lipid (%)	Ash (%)	Mg Contents (mg/kg)	Mg Retention (%)
0	70.32 ± 1.25	14.48 ± 0.72 ^b^	10.81 ± 0.66	5.44 ± 0.29	1043.61 ± 13.48 ^c^	1013.21 ± 221.45 ^a^
60	71.53 ± 1.09	16.20 ± 0.06 ^ab^	11.53 ± 0.29	4.56 ± 0.27	1333.10 ± 43.79 ^a^	325.64 ± 26.88 ^b^
120	72.07 ± 1.81	17.70 ± 0.58 ^a^	7.81 ± 0.41	5.26 ± 0.40	1366.26 ± 47.43 ^a^	133.11 ± 14.19 ^b^
240	71.09 ± 1.79	15.40 ± 0.58 ^ab^	8.51 ± 1.55	5.28 ± 0.12	1229.08 ± 16.62 ^ab^	59.55 ± 7.12 ^b^
480	70.67 ± 0.59	16.01 ± 0.41 ^ab^	9.76 ± 0.73	5.35 ± 0.47	1228.01 ± 12.05 ^ab^	23.51 ± 0.76 ^b^
960	70.95 ± 0.96	16.52 ± 0.21 ^ab^	9.69 ± 0.94	4.39 ± 0.24	1290.08 ± 32.85 ^ab^	21.47 ± 1.65 ^b^
1920	71.36 ± 2.20	15.66 ± 0.26 ^ab^	10.02 ± 1.86	4.37 ± 0.51	1162.20 ± 14.63 ^bc^	5.79 ± 0.98 ^b^
Polynomial contrasts						
Linear	ns	ns	ns	ns	ns	***
Quadratic	ns	*	ns	ns	***	***
Cubic	ns	ns	ns	ns	***	*

Each data point represents the mean of four replicates. Means in the same line with different superscripts indicate significant difference (*p* < 0.05). * *p* < 0.05, *** *p* < 0.001, ns: no significance.

**Table 5:** Plasma metabolites of *M. amblycephala* fed the experimental diets.

Dietary MgO NPs Levels (mg/kg)	GLU (mmol/L)	GSP (umol/L)	TCHO (mmol/L)	TRIG (mmol/L)	Mg (mmol/L)
0	5.35 ± 0.60 ^a^	381.75 ± 21.07 ^a^	5.44 ± 0.29	3.23 ± 0.16 ^a^	1.21 ± 0.01 ^c^
60	5.05 ± 0.50 ^ab^	342.75 ± 11.40 ^a^	4.56 ± 0.27	2.26 ± 0.19 ^b^	1.25 ± 0.02 ^c^
120	3.67 ± 0.34 ^abc^	189.00 ± 3.85 ^b^	5.26 ± 0.40	1.96 ± 0.21 ^b^	1.38 ± 0.03 ^bc^
240	3.32 ± 0.28 ^bc^	197.00 ± 5.31 ^b^	5.28 ± 0.12	1.60 ± 0.23 ^b^	1.41 ± 0.03 ^bc^
480	5.13 ± 0.47 ^ab^	338.50 ± 49.12 ^a^	5.35 ± 0.47	2.06 ± 0.21 ^b^	1.48 ± 0.05 ^b^
960	5.28 ± 0.22 ^ab^	365.50 ± 28.61 ^a^	4.39 ± 0.24	2.29 ± 0.23 ^b^	1.75 ± 0.09 ^a^
1920	2.78 ± 0.72 ^c^	191.75 ± 8.64 ^b^	4.37 ± 0.51	1.58 ± 0.16 ^b^	1.41 ± 0.05 ^bc^
Polynomial contrasts					
Linear	ns	ns	ns	*	***
Quadratic	***	***	ns	ns	*
Cubic	ns	ns	ns	ns	**

GLU, glucose; GSP, glycated serum proteins; TCHO, total cholesterol; TRIG, triacylglycerol. Each data point represents the mean of four replicates. Means in the same line with different superscripts indicate significant difference (*p* < 0.05). * *p* < 0.05, ** *p* < 0.01, *** *p* < 0.001, ns: no significance.

## Data Availability

The data generated during the current study are available from the first author.
